# Direct oral anticoagulants increase bleeding risk after endoscopic sphincterotomy: a retrospective study

**DOI:** 10.1186/s12876-021-01980-6

**Published:** 2021-10-24

**Authors:** Sakue Masuda, Kazuya Koizumi, Takashi Nishino, Tomohiko Tazawa, Karen Kimura, Junichi Tasaki, Chikamasa Ichita, Akiko Sasaki, Makoto Kako, Haruki Uojima, Ayumu Sugitani

**Affiliations:** 1 Department of Gastroenterology Medicine Center, Shonan Kamakura General Hospital, 1370-1 Okamoto, Kamakura, Kanagawa 247-8533 Japan; 2 Department of Gastroenterology, Internal Medicine, Kitasato University School of Medicine, Sagamihara, Kanagawa 252-0375 Japan; 3 Department of the Institute of Biomedical Research, Sapporo Higashi Tokushukai Hospital, Sapporo, Hokkaido 065-0033 Japan

**Keywords:** Anticoagulants, Direct oral anticoagulants, Endoscopy, Endoscopic sphincterotomy bleeding, Guidelines

## Abstract

**Background:**

Bleeding can be a serious adverse event of endoscopic sphincterotomy (EST). However, the risk of EST bleeding between direct oral anticoagulant (DOAC) users and those who received no antithrombotic agents has not been clarified. This study analyzed the risk factors for bleeding after EST in patients on DOAC and evaluated the Japan Gastroenterological Endoscopy Society (JGES) guidelines for gastroenterological endoscopy in patients undergoing antithrombotic treatment.

**Methods:**

We retrospectively analyzed 524 patients treated with EST who received DOAC or no antithrombotic drug from May 2016 to August 2019. We investigated the risk factors for bleeding. DOAC was typically discontinued for ≤ 1-day based on the JGES guideline. Although DOAC therapy recommenced the next morning after EST in principle, the duration of DOAC cessation and heparin replacement were determined by the attending physician based on each patient’s status.

**Results:**

The number of patients on DOAC (DOAC group) and those not on antithrombotic drug (no-drug group) was 42 (8.0%) and 482 (92.0%), respectively. DOAC was discontinued for ≤ 1-day in 17 (40.0%) patients and for > 1-day in 25 (60.0%). Of the 524 patients, 21 (4.0%) had EST bleeding. The bleeding rate was higher in the DOAC group (14.0%) (*p* = 0.004). Multivariate analysis showed that bleeding occurred more frequently in patients on DOAC (odds ratio [OR] 3.95, 95% confidence interval [CI] 1.37–11.4, *p* = 0.011), patients with low platelet counts (< 100,000/µl) (OR 6.74, 95% CI 2.1–21.6, *p* = 0.001), and elderly patients (> 80 years old) (OR 3.36, 95%CI 1.17–9.65, *p* = 0.024).

**Conclusions:**

DOAC treatment, low platelet count, and old age (> 80 years old) are risk factors for EST bleeding. Although the bleeding incidence increased in patients on DOAC who received antithrombotic therapy according to the JGES guidelines, successful hemostasis was achieved with endoscopy in all cases, and no thrombotic events occurred after cessation of DOAC. Thus, the JGES guidelines are acceptable.

## Background

Endoscopic sphincterotomy (EST) is an essential procedure in endoscopic retrograde cholangiopancreatography (ERCP). However, bleeding is a potential complication of EST. The rate of bleeding associated with EST is 1–5% [1–7]. The rates of severe bleeding associated with EST are statistically significantly higher among anticoagulant users than among non-users [8]; however, the risk of EST bleeding is lower with direct oral anticoagulant (DOAC) use than with warfarin use [9, 10]. Moreover, the risk of EST bleeding between DOAC users and those who received no antithrombotic agents has not been clarified.

Recently, a guideline on gastroenterological endoscopy in patients undergoing antithrombotic treatment was published by the Japan Gastroenterological Endoscopy Society (JGES) [11, 12]. However, the evidence levels for several items in the guidelines are low, and the guidelines still need to be verified in clinical settings [12]. In addition, balancing the risk of bleeding against that of thromboembolism is difficult in patients in whom DOAC has been discontinued [13, 14]. In this study, we aim to investigate the risk factors for bleeding after EST and evaluate the JGES guidelines for gastroenterological endoscopy in patients receiving DOAC.

## Methods

The study was reviewed and approved by the Future Medical Research Center Ethical Committee’s institutional review board (IRB No. TGE00934-024). All procedures have been performed in accordance with the ethical standards laid down in the 1964 Declaration of Helsinki and its later amendments.

### Study population

This retrospective study was conducted at Shonan Kamakura General Hospital in Japan. Study enrollment commenced in May 2016 and ended at the end of August 2019. This study included patients undergoing EST who received DOAC prior to EST and those who received no antithrombotic agent. Patients on warfarin or antiplatelet therapy were excluded. We investigated the patients’ characteristics, ERCP findings, incidence of EST bleeding, risk factors for bleeding, and DOAC cessation period. Under the conditions of α = 0.05 and β = 0.20, according to the EZR statistical software, the sample size required 39 patients on DOAC and 448 patients not receiving antithrombotic drugs.

**Fig. 1:**
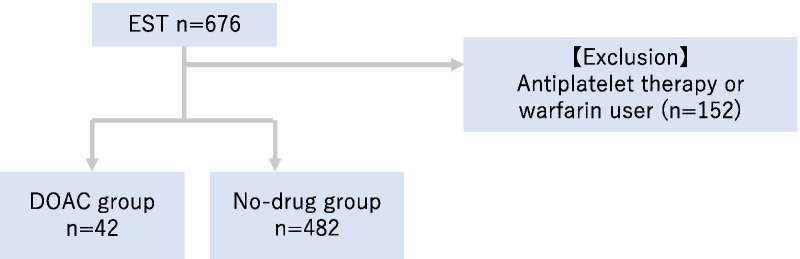
Study population. Of the 676 patients treated with EST from May 2016 to August 2019, 152 patients were excluded based on the exclusion criteria (this study enrolled patients undergoing EST who were treated with either a DOAC prior to the EST or no antithrombotic agents). The exclusion criterion was the use of warfarin or antiplatelet therapy only. EST, endoscopic sphincterotomy; DOAC, direct oral anticoagulants

Of the 676 patients enrolled, 152 patients excluded based on the exclusion criterion. A total of 524 patients were finally included in the analysis (Fig. 1). Moreover, the number of patients on DOAC treatment (DOAC group) was 42 (8.0%) and that of those on no antithrombotic drug (no-drug group) was 482 (92.0%).

### Endoscopic procedure

EST was basically performed with a pull-type sphincterotome (Clever Cut V3; Olympus, Tokyo, Japan) through a side-viewing endoscope (JF-260V, TJF-260V; Olympus, Tokyo, Japan). EST was performed by experts who had performed > 1000 ERCP procedures. Sphincterotomy was performed in the 11–12 o’clock direction, and an electrosurgical unit (ERBE ICC200; Surgical Technology Group, Hampshire, England, UK) was put in ENDOCUT mode; a 120-W power setting was employed. Medium EST, which extends from one third to two thirds of the total length of the ampulla, was performed in most cases. Small EST, which was within one third of the total length of the ampulla, was occasionally used (i.e., in patients with a potential risk of perforation such as those in whom oral protraction of the ampulla was small). In patients in whom biliary cannulation was difficult, precut using a needle-type sphincterotome (KD-10Q-1; Olympus, Tokyo, Japan) or a transpancreatic precut sphincterotomy was performed. The diameter of the EPLBD balloon (CRE, Boston Scientific Japan, Tokyo, Japan) (GIGA2, Kaneka corporation, Tokyo, Japan) was selected to correspond to the diameter of the distal bile duct. After EPLBD balloon insertion into the papilla, the balloon was gradually pressurized until waist disappearance.

### Antithrombotic agents

In this study, DOAC was typically discontinued for ≤ 1-day based on the JGES guideline. Although DOAC therapy recommenced the next morning after EST in principle, the duration of DOAC cessation and heparin replacement were determined by the attending physician based on each patient’s status. The most commonly used heparin-bridging technique before EST was the replacement of oral anticoagulants with unfractionated heparin 2–3 days after admission, with dose adjustments to attain the required activated partial thromboplastin time (APTT) [11]. Heparin administration was stopped 4–6 h before EST and restarted 4–6 h after EST.

### Definitions

EST bleeding was defined as bleeding during or after EST. Bleeding during EST was described as pulsatile bleeding or bleeding that continued at the end of planned procedures such as lithotripsy or stent replacement; bleeding after EST was defined as hematemesis, bloody stool, and bleeding that are not due to other causes of gastrointestinal bleeding confirmed by endoscopy within 2 weeks post-EST*.*

Hemostatic procedures, such as balloon compression and hypertonic saline epinephrine solution (HSE) administration, were indicated in patients whose point of bleeding could not be identified. After achieving hemostasis, additional procedures such as coagulation or hemoclipping were performed when the bleeding point was identified. In patients whose point of bleeding could be identified, coagulation using electrical devices, was performed for oozing bleeding and hemoclipping for spurting bleeding or exposed vessels.

Low platelet count was defined as < 100,000/µl, which is a severe cholangitis classification in the Tokyo Guideline 2018 of cholangitis. Moreover, several studies also defined low platelet count as < 100,000/µl [10, 15]. An elderly patient was defined as one aged > 80 years, following the definition of Muro et al., who reported that this age group is at risk for EST bleeding [10].

Renal dysfunction was defined as eGFR (estimated glomerular filtration rate) (ml/min/1.73 m^2^) < 60.

### Statistical analysis

The Mann–Whitney U-test was used to compare continuous variables, which were non-normally distributed, and χ^2^-test or Fisher’s exact test was used to compare categorical variables. A multivariate analysis was performed using logistic regression. Two-tailed *p* values < 0.05 were considered statistically significant. All statistical analyses were performed with EZR (Saitama Medical Center, Jichi Medical University, Saitama, Japan), which is a graphical user interface for R (The R Foundation for Statistical Computing, Vienna, Austria). More precisely, it is a modified version of R commander and was designed to allow additional statistical functions that are frequently used in biostatistics [16].

## Results

### Patient characteristics

The characteristics of the patients in the DOAC and no-drug groups are shown in Table 1.

**Table 1:** Patient characteristics

	DOAC group	No-drug group	*p* value
	n = 42 (8.0%)	n = 482 (92.0%)	
Sex	Male 23, female 19	Male 234, female 248	0.52
Age	82 (65–95) years	76 (25–106) years	0.001
Indication for ERCP			
Malignant biliary stricture	10 (24.0%)	108 (22.4%)	0.848
Bile duct stone	31 (74.0%)	333 (69.1%)	0.603
Cholecystitis	1 (2.0%)	22 (4.6%)	0.99
Acute pancreatitis	0	2 (0.4%)	0.99
Chronic pancreatitis	0	4 (0.8%)	0.99
Others	0	13 (2.7%)	0.613
Underlying disease^‡^			
Total	24 (57.0%)	47 (9.8%)	< 0.001
Hemodialysis	0	5 (1.0%)	0.99
Liver cirrhosis	3 (7.0%)	6 (1.2%)	0.029
Cardiovascular disease	13 (31.0%)	25 (5.1%)	< 0.001
Cerebral hemorrhage	1 (2.0%)	9 (1.9%)	0.57
Stroke	13 (31.0%)	10 (2.1%)	< 0.001
Cholangitis	33 (79.0%)	314 (65.1%)	0.09
Platelet (µl)	17.1 (8.0–52.4)	20.5 (4.5–67.8)	0.013
PT-INR	1.21 (1.02–1.72)	1.06 (0.81–2.46)	< 0.001
APTT	35.9 (26.0–62.6)	29.8 (3.9–75.8)	< 0.001
eGFR	63.5 (15.4–101.5)		
Number of patients with eGFR < 60	20 (48.0%)		
Patients with eGFR < 60 who were receiving a reduced dose of DOAC	16/20 (80.0%)		

*APTT* Activated partial thromboplastin time, *DOAC* direct oral anticoagulant, *ERCP* endoscopic retrograde cholangiopancreatography, *PT-INR* prothrombin time-international normalized ratio, *eGFR* estimated glomerular filtration rate

^‡^There are some duplicates in each group

In the DOAC group, the median age was 82 (range 65–95) years and the male-to-female ratio was 1.21. The indications for EST were malignant stricture in 10 (24.0%) patients, bile duct stone in 31 (74.0%), and acute cholecystitis in 1 (2.0%). Underlying diseases were present in 24 (57.0%) patients, including cardiovascular disease in 13 (31.0%), liver cirrhosis in 3 (7.0%), stroke in 13 (31.0%), and cerebral hemorrhage in 1 (2.0%). Moreover, cholangitis was found in 33 (79.0%) patients. The median platelet count, prothrombin time international normalized ratio (PT-INR) and APTT were 17.1 × 10^4^/µl (range 8.0–52.4 × 10^4^/µl), 1.21 (range 1.02–1.72), and 35.9 (range 26.0–62.6) seconds, respectively. The median eGFR was 63.5 (range 15.4−101.5). The number of patients with eGFR < 60 was 20 (48.0%). Patients with renal dysfunction who were receiving a reduced dose of DOAC were 16 (80.0%) out of 20. Although, in principle, DOAC therapy was restarted the morning after EST, the length of DOAC cessation was determined by the attending physician according to the patient’s condition, resulting in resumption of DOAC therapy the morning after EST, in 35 patients (83.0%).

In the no-drug group, the median age was 76 (range 25–106) years, and the male-to-female ratio was 0.94. The indications for EST were malignant stricture in 108 (22.4%), bile duct stone in 333 (69.1%), acute cholecystitis in 22 (4.6%), acute pancreatitis in 2 (0.4%), and chronic pancreatitis in 4 (0.8%) patients. Underlying diseases were noted in 47 (9.8%) patients, including cardiovascular disease in 25 (5.1%), hemodialysis in 5 (1.0%), liver cirrhosis in 6 (1.2%), stroke in 10 (2.1%), and cerebral hemorrhage in 9 (1.9%). Cholangitis was observed in 314 (65.1%) patients. The median platelet count, PT-INR, and APTT were 20.5 × 10^4^/µl (range 4.5–67.8 × 10^4^/µl), 1.06 (range 0.81–2.46), and 29.8 (range 3.9–75.8) seconds, respectively.

As shown in the *p*-values in Table 1, age (*p* = 0.001), incidence of cardiovascular disease (*p* < 0.001), stroke (*p* < 0.001), or liver cirrhosis (*p* = 0.029), PT-INR (*p* < 0.001), and APTT (*p* < 0.001) were higher in the DOAC group than in the no-drug group; the platelet count (*p* = 0.013) was lower in the DOAC group than in the no-drug group.

### ERCP findings

In the DOAC group, periampullary diverticulum was found in 13 (31.0%) patients. Precut was performed in 1 (2.0%) patient, lithotripsy in 33 (79.0%), and endoscopic papillary balloon dilation (EPLBD) in 5 (12.0%). A self-expandable metallic stent (SEMS) was implanted in 2 (5.0%) patients and plastic stent in 5 (12.0%); endoscopic nasobiliary drainage tube (ENBD) was inserted in 3 (7.0%) patients. Complications were noted in 7 (17.0%) patients, including bleeding in 6 (14.0%) and acute cholecystitis in 1 (2.0%). DOACs were replaced with heparin in 12 (29.0%) patients. Median APTT before EST in patients with heparin replacement was 39.6 (range 31.0–62.6).

In the no-drug group, periampullary diverticulum was observed in 134 (27.8%) patients. Precut was performed in 4 (0.8%) patients, lithotripsy in 345 (71.6%), and EPLBD in 25 (5.2%). SEMS was implanted in 50 (10.4%) patients and plastic stent in 100 (20.7%); ENBD was inserted in 28 (5.8%) patients. Complications were noted in 43 (8.9%) patients, including bleeding in 15 (3.1%), pancreatitis in 17 (3.5%), perforation in 3 (0.6%), acute cholecystitis in 5 (1.0%), and other complications in 4 (0.8%).

Only bleeding was higher in the DOAC group than the no-drug group (*p* = 0.004). Table 2 shows the ERCP findings.

**Table 2:** Endoscopic retrograde cholangiopancreatography findings

	DOAC group	No-drug group	*p* value
	n = 42 (8.0%)	n = 482 (92.0%)	
Periampullary diverticulum	13 (31.0%)	134 (27.8%)	0.72
Precut	1 (2.0%)	4 (0.8%)	0.343
Lithotripsy	33 (79.0%)	345 (71.6%)	0.375
EPLBD	5 (12.0%)	25 (5.2%)	0.082
Metallic stent	2 (5.0%)	50 (10.4%)	0.415
Plastic stent	5 (12.0%)	100 (20.7%)	0.227
ENBD	3 (7.0%)	28 (5.8%)	0.729
Complication^‡^			
Bleeding	6 (14.0%)	15 (3.1%)	0.004
Pancreatitis	0	17 (3.5%)	0.384
Perforation	0	3 (0.6%)	0.99
Cholecystitis	1 (2.0%)	5 (1.0%)	0.396
Others	0	4 (0.8%)	0.99
Total	7 (17.0%)	43 (8.9%)	0.104
Heparin replacement	12 (29.0%)	0	< 0.001
APTT before EST in patients with heparin replacement	39.6 (31.0–62.6)		

*DOAC* Direct oral anticoagulant, *ENBD* endoscopic nasobiliary drainage, *EPLBD* endoscopic papillary large balloon dilation, *APTT* activated partial thromboplastin time

^‡^There are some duplicates in each group

### EST bleeding and outcomes

Of 524 patients, 21 (4.0%) had bleeding. The bleeding rate in the DOAC group (14.0%, 6/42) was statistically significantly higher than that in the no-drug group (3.0%, 15/482) (*p* = 0.004) (Table 3). All bleeding patients in the DOAC group had normal renal function, and there were no patients with “eGFR < 60 without DOAC dose reduction” in the bleeding group.

**Table 3:** Endoscopic sphincterotomy bleeding and outcomes

	DOAC group n = 42	No-drug group n = 482	*p* value
Bleeding	6 (14.0%)	15 (3.1%)	0.004
Bleeding during EST	2/6 (33.0%)	8/15 (53.3%)	0.635
Post-EST bleeding	4/6 (67.0%)	7/15 (46.7%)	0.635
Shock	2/6 (33.0%)	2/15 (13.3%)	0.544
Transfusion	1/6 (17.0%)	3/15 (20.0%)	0.99
Change in hemoglobin level	− 0.65 (− 7.3 to 0.7)	− 0.9 (− 5.1 to 1.7)	0.134
Hospitalization	8 (4–65) days	7 (3–71) days	0.06

Data are presented as median (range)

*DOAC* Direct oral anticoagulant, *EST* endoscopic sphincterotomy

In the DOAC group, bleeding occurred during ERCP in 2 (33.0%) patients and after ERCP in 4 (67.0%) patients. The incidence of shock was 33.0% (2/6); 17.0% (1/6) of the patients needed transfusion. The median change in hemoglobin level was − 0.65 (range − 7.3 to 0.7) g/dl. The change in hemoglobin level was defined as a change within 1 week after ERCP. The median duration of hospitalization was 8 (range 4–65) days.

In the no-drug group, bleeding was noted during ERCP in 8 (53.3%) patients and after ERCP in 7 (46.7%) patients. The incidence of shock was 13.3% (2/15), and transfusion was needed in 20.0% (3/15) of the patients. The median change in hemoglobin level was − 0.9 (range − 5.1 to 1.7) g/dl, and the median duration of hospitalization was 7 (range 3–71) days. No significant differences were found between the two groups.

**Fig. 2:**
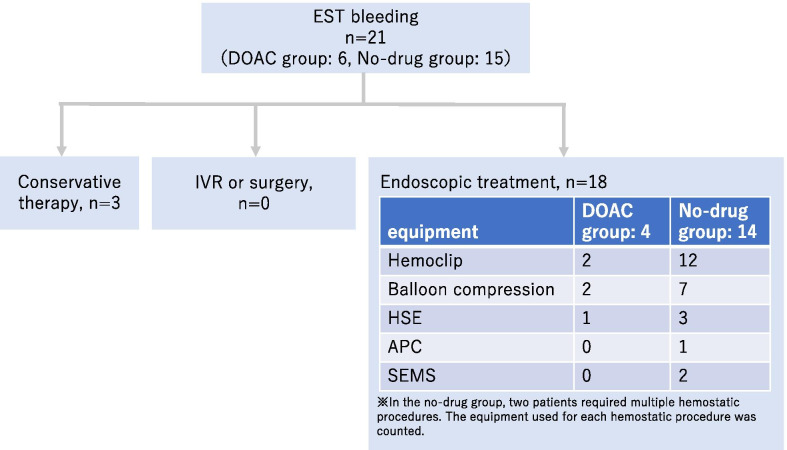
Interventions for endoscopic sphincterotomy bleeding. Hemostasis was achieved with endoscopy or conservative therapy. Interventional radiology or surgery was not necessary. In the no-drug group, two patients required multiple hemostatic procedures, but there was no significant difference in the number of procedures. APC: argon plasma coagulation; HSE: hypertonic saline-epinephrine; SEMS: self-expandable metallic stent

All patients with EST bleeding were successfully treated with endoscopy or conservative therapy. Interventional radiology or surgery was not needed to achieve hemostasis. In the no-drug group, two patients required multiple hemostatic procedures, one required two procedures and the other, four. However, there was no significant difference in the number of procedures between the two groups (*p* = 0.99) (Fig. 2).

### Multivariate analysis of risk factors for bleeding after EST (Table 4)

**Table 4:** Multivariate analysis of risk factors for bleeding after endoscopic sphincterotomy

Multivariate analysis	Bleeding n = 21	No bleeding n = 503	Univariate analysis, *p* value	Multivariate analysis, *p* value	Odds ratio	95% CI
Age > 80 years	16 (76.0%)	205 (40.8%)	0.002	0.024	3.36	1.17–9.65
Underlying disease						
Cardiovascular disease	1 (5.0%)	37 (7.4%)	0.99			
Stroke	1 (5.0%)	22 (4.4%)	0.99			
Liver cirrhosis	0	9 (1.8%)	0.99			
DOAC use	6 (29.0%)	36 (7.2%)	0.004	0.011	3.95	1.37–11.4
Combination of DOAC and antiplatelet drugs	2 (10.0%)	10 (2.0%)	0.079			
Platelet count < 100,000/µl	5 (24.0%)	17 (3.4%)	0.001	0.001	6.74	2.1–21.6
ERCP findings						
Periampullary diverticulum	7 (33.0%)	140 (27.8%)	0.622			
Precut	1 (5.0%)	4 (0.8%)	0.186			
Lithotripsy	14 (67.0%)	364 (72.4%)	0.62			
EPLBD	3 (14.0%)	27 (5.4%)	0.112			
SEMS	3 (14.0%)	49 (9.7%)	0.453			

Area under the ROC curve: 0.748; Multicollinearity: < 5

*CI* Confidence interval, *DOAC* direct oral anticoagulant, *EPLBD* endoscopic papillary large balloon dilation, *ENBD* endoscopic naso-biliary drainage, *SEMS* self-expandable metallic stent, *ROC* receiver-operating characteristic

Factors that were considered clinically significant were included in the multivariate analysis. INR and APTT were difficult to interpret clinically because of the variable effects of DOAC on INR and APTT; thus, INR and APTT were not included in the analysis. Hemodialysis was considered not to be involved in EST bleeding in this study since there were only 5 patients who had no bleeding. Finally, Age > 80 years, DOAC use, and platelet count < 100,000/µl were included in the multivariate analysis. Multivariate analysis showed that bleeding occurs more frequently in patients on DOAC (odds ratio [OR] 3.95, 95% confidence interval [CI] 1.37–11.4, *p* = 0.011), those with low platelet count (< 100,000/µl) (OR 6.74, 95% CI 2.1–21.6, *p* = 0.001), and elderly patients (> 80 years old) (OR 3.36, 95% CI 1.17–9.65, *p* = 0.024). In this model, the area under the ROC curve was 0.748, and multicollinearity was < 5.

In this study, of the 22 patients with low platelet count (< 100,000/µl), 1 (5.0%) had liver cirrhosis, 1 (5.0%) had aplastic anemia, and 20 (91.0%) had cholangitis. Of the 20 patients with cholangitis, 17 (85.0%) showed improvement in platelet count as well as in cholangitis.

### DOAC cessation period

Multivariate analysis revealed that DOAC is a risk factor for bleeding; hence, we examined the length of DOAC cessation (Table 5). Forty-two patients on DOAC were divided into two groups according to the number of days DOAC was discontinued, that is, ≤ 1-day (n = 17, 40.0%) and > 1-day (n = 25; 60.0%).

**Table 5:** Direct oral anticoagulant cessation period

	≤ 1-day cessation	> 1-day cessation	*p* value
	n = 17	n = 25	
EST bleeding	5 (29.0%)	1 (4.0%)	0.032
Age > 80 years	15 (88.0%)	14 (56.2%)	0.041
Underlying disease^‡^			
Hemodialysis	0	0	1
Cardiovascular disease	4 (24.0%)	9 (36.0%)	0.505
Stroke	4 (24.0%)	9 (36.0%)	0.505
Liver cirrhosis	3 (18.0%)	0	0.059
Combination of antiplatelet drugs	6 (35.0%)	6 (24.0%)	0.498
Heparin replacement	0	12 (48.0%)	< 0.001
APTT before EST in patients with heparin replacement		39.6 (31.0–62.6)	
Platelet count < 100,000/µl	2 (12.0%)	1 (4.0%)	0.556
eGFR	66.5 (31.3–101.5)	57.9 (16.4–94.2)	0.187
The number of patients with eGFR < 60	5 (29.0%)	15 (60.0%)	0.066
Patients with eGFR < 60 who were receiving a reduced dose of DOAC	3/5 (60.0%)	13/15 (86.7%)	0.249
ERCP findings			
Periampullary diverticulum	4 (24.0%)	9 (36.0%)	0.505
Precut	0	1 (4.0%)	0.99
Lithotripsy	14 (82.0%)	18 (72.0%)	0.49
EPLBD	1 (6.0%)	4 (16.0%)	0.632
ENBD	2 (12.0%)	1 (4.0%)	0.556
Thromboembolism	0	0	1

*ENBD* Endoscopic naso-biliary drainage, *EPLBD* endoscopic papillary large balloon dilation, *EST* endoscopic sphincterotomy, *APTT* activated partial thromboplastin time, *eGFR* estimated glomerular filtration rate

In the ≤ 1-day group, bleeding occurred in 5 (29.0%) patients, and 15 (88.0%) were elderly patients (> 80 years old). Underlying comorbidities, were cardiovascular disease in 4 (24.0%), stroke in 4 (24.0%), and liver cirrhosis in 3 (18.0%) patients. Six (35.0%) patients used a combination of antiplatelet drugs and DOAC, 0 had heparin replacement, and 2 (12.0%) had a platelet count < 100,000. Antiplatelet drug users included 4 aspirin, 1 thienopyridines, and 1 both. All four patients on aspirin were continued on aspirin. Thienopyridines were replaced with aspirin in the patient on thienopyridines. In the patient on a combination of aspirin and thienopyridines, aspirin was continued and thienopyridines discontinued 5 days before EST. In cases where antiplatelet drugs were discontinued or replaced, on principle, they were reinstated at the same time as DOACs. There were no cases of heparin replacement. The median eGFR was 66.5 (range 31.3–101.5). The number of patients with eGFR < 60 was 5 (29.0%). Patients with renal dysfunction who were receiving a reduced dose of DOAC were 3 of 5 (60.0%). Periampullary diverticulum was found in 4 (24.0%) patients. Precut was not performed in any patient in this group, whereas lithotripsy was performed in 14 (82.0%) patients and EPLBD in 1 (6.0%). ENBD was inserted in 2 (12.0%) patients.

In the > 1-day group, bleeding was noted in 1 (4.0%) patient, and 14 (56.0%) were elderly patients (> 80 years old). Underlying comorbidities, were cardiovascular disease in 9 (36.0%) patients, stroke in 9 (36.0%), and liver cirrhosis in 0. Six (24.0%) patients received a combination of antiplatelet drugs and DOAC, 12 (48.0%) had heparin replacement, and 1 (4.0%) had a platelet count < 100,000. Antiplatelet drug users included 6 aspirin. All 6 were continued on aspirin. Median APTT before EST in patients with heparin replacement was 39.6 (range 31.0–62.6). The median eGFR was 57.9 (range 16.4–94.2). The number of patients with eGFR < 60 was 15 (60.0%). Patients with renal dysfunction who were receiving a reduced dose of DOAC was 13 of 15 (87.0%). Periampullary diverticulum was observed in 9 (36.0%) patients. Precut was performed in 1 (4.0%), lithotripsy in 18 (72.0%), and EPLBD in 4 (16.0%) patients; ENBD was inserted in 1 (4.0%) patient.

As shown in the *p*-values in Table 5, the number of EST bleeding (*p* = 0.032) and elderly patients (*p* = 0.041) were higher in the ≤ 1-day group than the > 1-day group. Heparin was used only in the > 1-day group. There was no EST bleeding in patients who had heparin replacement or renal dysfunction. No thrombotic events occurred during hospitalization.

## Discussion

Several single-center studies have analyzed the risk factors for EST bleeding [3, 15, 17–19]. Although most of these reports only included patients treated with warfarin, a few recent reports analyzed the risk factors for EST bleeding in patients treated with DOAC [9, 10]. However, these reports compared the risk of EST bleeding between DOAC and warfarin users; patients not receiving anticoagulants were not considered. As the population ages and the incidence of chronic disease rises, the need for anticoagulants also increases. Particularly, the use of DOAC has increased recently. Thus, the risk factors for bleeding after EST in patients on DOAC were analyzed and compared with those of patients not receiving antithrombotic therapy.

Previous studies have shown that the rate of bleeding associated with EST is 1–5% [1–7]. In this study, the rate of EST bleeding was 4.0% (21/524), and the bleeding rate in the DOAC group (14.0%, 6/42) was significantly higher than that in the no-drug group (3.1%, 15/482). While the bleeding rate in the DOAC group in this study was higher than that in previous studies, it was comparable in all cases, to those of other reports [9, 10]. In addition, multivariate analysis revealed that the significant risk factors for EST bleeding were DOAC, low platelet count (< 100,000/µl), and old age (> 80 years old). Thus, the rate of EST bleeding in the DOAC group in our study is reliable. Moreover, among the studies that investigated the risk factors for EST bleeding [2, 20, 21], no report revealed the relationship between platelet count and EST bleeding. In our study, 20 of the 22 patients with platelet counts < 100,000 had cholangitis, and 17 (85.0%) of these patients showed improvement in platelet count as well as in cholangitis, suggesting that the main cause of low platelet count was cholangitis. Some reports described heparin replacement as a risk factor for post-EST bleeding [9, 10, 15]. However, in our study, there was no EST bleeding with heparin replacement. Therefore, we conclude that heparin was not related to EST bleeding in our study.

A previous study demonstrated that the rate of severe bleeding associated with EST among anticoagulant users was statistically significantly higher than that among non-users [8]. In our study, although the EST bleeding rate was higher in the DOAC group than the no-drug group, no significant differences in the extent of bleeding based on the rate of shock, necessity of transfusion, and change in hemoglobin level were found between the groups. In a recent study, the risk of EST bleeding was lower in DOAC users than in warfarin users [9, 10].

Our evaluation of the DOAC cessation period showed that the incidence of EST bleeding was significant higher in the ≤ 1-day group than the > 1-day group. Therefore, to decrease the risk of EST bleeding in the DOAC group, a longer period of DOAC treatment cessation may be necessary; it may, however, also increase the risk of thromboembolic events [22].

The incidence rate of thromboembolic events after temporary warfarin cessation was 0.7% in a large prospective cohort study that enrolled 6761 patients with 1293 episodes of anticoagulation interruption [23]. In another study, the incidence rate of thromboembolic events after temporary warfarin cessation was 4.2% (4/96) [22]. Moreover, cessation of anticoagulant therapy for a long period (> 48 h) was associated with thromboembolic events. Therefore, in patients at risk of thromboembolic events, early resumption of anticoagulant therapy after EST, i.e., within 48 h, is recommended [22]. Our study followed the JGES guideline for antithrombotic therapy, and no thrombotic events were observed during hospitalization.

Various endoscopic approaches for the treatment of EST bleeding have been reported, including injection therapy with HSE [24], balloon compression [25], argon plasma coagulation [26], and hemoclip [27]. Although post-EST bleeding is not associated with increased mortality and morbidity rates, length of hospital stay as well as costs may increase [2]. In our study, all patients with EST bleeding were successfully treated with endoscopic hemostasis or conservative therapy. No significant differences in the length of hospital stay between the DOAC and no-drug groups were found. Hence, the JGES guideline was acceptable.

This study has several limitations. The study population was small. In addition, this was a single tertiary referral center retrospective study; thus, some uncontrolled confounding factors that affected the results possibly exist. Given these limitations, a prospective randomized multicenter trial may be warranted to standardize the approach for DOAC cessation, although these and other data are quite convincing, questioning the need for a true randomized study.

## Conclusion

Caution needs to be exercised when caring for patients on DOAC, those with low platelet count, and elderly patients after EST, as the occurrence of bleeding is more frequent in these populations. A longer DOAC cessation period may be necessary in the DOAC group to achieve the same bleeding rate as that of the no-drug group; nevertheless, this may result in thromboembolic events. Our study showed that hemostasis was achieved in all patients with EST bleeding and that no thromboembolic events occurred. Therefore, although the bleeding rate was higher in the DOAC group than the no-drug group, EST based on short-term DOAC cessation according to the JGES guideline was considered valid because the prevention of thromboembolic event is as important as the achievement of hemostasis. Further studies are required to broaden our understanding of DOACs, which are increasingly being used in clinical settings, including a study on how the cessation period of DOAC relates to renal function.

## Data Availability

The technical appendix, statistical code, and dataset are available from the corresponding author upon request. No additional data are available.

## Abbreviations

EST: Endoscopic sphincterotomy
DOAC: Direct oral anticoagulant
JGES: The Japan Gastroenterological Endoscopy Society
ERCP: Endoscopic retrograde cholangiopancreatography
HSE: Hypertonic saline epinephrine solution
PT-INR: The median prothrombin time international normalized ratio (PT-INR)
APTT: The median activated partial thromboplastin time
EPLBD: Endoscopic papillary balloon dilation
SEMS: Self-expandable metallic stent
ENBD: Endoscopic nasobiliary drainage tube
